# Practical Echocardiographic Approach of the Regurgitant Mitral Valve Assessment

**DOI:** 10.3390/diagnostics12071717

**Published:** 2022-07-15

**Authors:** Rebeca Muñoz-Rodríguez, María Amelia Duque-González, Aida Tindaya Igareta-Herraiz, Mauro Di Silvestre, María Manuela Izquierdo-Gómez, Flor Baeza-Garzón, Antonio Barragán-Acea, Francisco Bosa-Ojeda, Juan Lacalzada-Almeida

**Affiliations:** Cardiology Unit, University Hospital of the Canary Islands, Tenerife, 38320 San Cristóbal de La Laguna, Spain; mameliaduque@gmail.com (M.A.D.-G.); aidatindaya@hotmail.com (A.T.I.-H.); jibjab547@gmail.com (M.D.S.); mmizqgom@gmail.com (M.M.I.-G.); flor-85@hotmail.es (F.B.-G.); barraganacea@gmail.com (A.B.-A.); franbosa@ull.edu.es (F.B.-O.); jlacalzada@gmail.com (J.L.-A.)

**Keywords:** mitral regurgitation, mitral valve, severity, Carpentier, echocardiography

## Abstract

Mitral regurgitation is the second-most frequent valvular heart disease in Europe after degenerative aortic stenosis. It is associated with significant morbidity and mortality, and its prevalence is expected to increase with population aging. Echocardiography is the first diagnostic approach to assess its severity, constituting a challenging process in which a multimodality evaluation, integrating quantitative, semiquantitative and qualitative methods, as well as a detailed evaluation of the morphology and function of both left ventricle and atria is the key. In this review, we would like to provide a practical diagnosis approach on the mitral valve regurgitation mechanism, severity quantification, and planning of future therapeutic options.

## 1. Introduction

Mitral regurgitation (MR) is the second-most frequent valvular heart disease in Europe after degenerative aortic stenosis and affects more than 2 million people in the USA. Associated with significant morbidity and mortality, its prevalence is expected to increase with population aging. This valve disorder progresses insidiously due to heart-compensatory mechanisms. Mortality rates per year are estimated around a 3% in people over 50 years old with moderate regurgitating and 6% when the valve disfunction is quantified as severe [1].

The mitral valve is a complex anatomical structure whose physiological functioning relies on the biomechanical properties and structural integrity of its components. Their compromise can lead to mitral valve dysfunction [2]. Assessing its morphology can reveal various normal and abnormal features, which can be associated with deteriorating clinical outcomes [3]. The mitral valve has a geometry comprising the mitral annulus, the anterior and posterior leaflets, and the subvalvular apparatus. The valve is obliquely located in the heart and has a close relation to the aortic valve. The leaflets are a continuous band of tissue extending from the annulus. According to their geometrical form and anatomical connection to the annulus, the leaflets are divided into anterior and posterior. The free edge of the mural leaflet is often divided into three or more scallops or segments described as lateral, middle, and medial or assigned terms such as P1, P2, and P3. Their size is variable [4].

Transthoracic echocardiography is the first diagnosis approach that should be performed to assess the valve disfunction, giving a complete morphological description of the structures, the regurgitant mechanism, and etiology [3]. In addition, the description of other cardiac structures morphology and function is necessary to make a correct evaluation of the severity of the valve disease and a pathophysiological integration, such as the dimension and remodeling of the left ventricle (LV) and left atria (LA), the filling pressures in both cavities (assessing the response of the volume overload), the existence of pulmonary hypertension, and the study of coexisting other valvular disorders. It is remarkable that the severity of MR can be variable, and its severity assessment approach is often performed suboptimally [5]. Although transthoracic echocardiography is the first-line diagnostic test, transesophageal ultrasound allows a more precise definition of morphology and severity, which is essential to guide transcatheter treatments and repair surgeries [6].

MR mechanism and proper severity assessment constitute a diagnostic challenge we must face every day. Given the mortality, morbidity, and the broad spectrum of therapeutic options from medical treatment to interventionism, it is elemental to perform an accurate evaluation.

## 2. Etiology and Regurgitant Mechanisms

The elucidation of the regurgitant mechanism has an impact on the consideration of future therapeutic options [3]. The first step in a correct filiation of MR is to classify it as primary, secondary, or mixed etiology. To perform an adequate evaluation, it is necessary to carefully analyze the morphology of the mitral apparatus (leaflets, subvalvular apparatus, annulus, and supporting myocardium) as well as their motion. Given the aging of the population, mixed etiology is likely to become more prevalent.

### 2.1. Primary Mitral Regurgitation

In this group, structural valvular damage is observed in the leaflets, chordae tendineae, annulus, or papillary muscles. The main cause of primary MR is the myxomatous degeneration of the leaflets resulting in valve prolapse. There is an important variability in the severity of myxomatous degeneration from fibroelastic deficiency to Barlow’s disease (commonly characterized by bileaflet prolapse). Other causes of primary MR are the leaflet perforation and cleft leaflets as well as connective tissue diseases, drugs, rheumatic diseases, or even radiation [7].

### 2.2. Secondary Mitral Regurgitation

The main characteristic of this group is the absence of structural damage of the leaflets and subvalvular apparatus, such as the MR subsidiary to LV geometry alterations, ventricular remodeling, inferobasal segmental disorders, or atrium morphology alterations.

Secondary MR due to alterations in the geometry of the LV should be differentiated into ischemic and non-ischemic etiology. The remodeling that occurs in both processes may have common characteristics; hence, both situations produce MR due to ventricular dilatation and lateral displacement of the papillary muscles, causing reduced valve closing forces and abnormally increased tethering forces of the leaflets to LV. This process is self-perpetuating, as MR leads to LV dilatation, which leads to more laterally papillary muscle displacement, annular dilatation, and then more MR. Apical displacement of the coaptation line within the LV is appreciated, conditioning an incomplete MV leaflet closure. It is essential to understand the global relationship between the LV, papillary muscle position, and leaflet motion and coaptation line to make an accurate etiological diagnosis [8,9].

The ischemic etiology should be suspected in the presence of inferior regional wall motion abnormalities (scars, dyskinesia, hypokinesia), which cause movement restriction in the posterior leaflet, generating the MR, whereas the non-ischemic is characterized for the restriction of both leaflets, LV dilatation, and displacement of both papillary muscles [10]. Commonly, when there is a restriction of the posterior leaflet, the jet is oriented ipso-laterally, pointing out the restricted leaflet. However, in case of prolapsing leaflets, the MR jets points out away from the diseased leaflet to the LA contra-laterally [3].

Situations such as the existence of a left bundle branch block, especially in patients with heart failure with reduced ejection fraction or right ventricular pacing, generate ventricular asynchrony, which causes a decrease in closing forces and dyssynchronous papillary muscle function, giving rise to the MR [11].

Another common cause of secondary MR is known as “functional atrial MR”, which occurs in patients with chronic atrial fibrillation with LA and/or right atria dilatation. In these circumstances, the LV is not dilated, but the annular dilation causes an increase in pressure in the LA that produces malcoaptation of the mitral leaflets [12].

### 2.3. Carpentier Classification

MR can be classified according to the Carpentier classification (Table 1), which facilitates the understanding of the etiology as well as therapeutic approach [13].

Carpentier classification type I is characterized by normal sized leaflets with normal motion. Valve annular dilatation or deformation can explain the MR as well as the presence of leaflet perforation (for instance, due to infective endocarditis) or congenital clefts. Moreover, the MR secondary to atria dilatation and non-ischemic cardiomyopathy are included in this group.

Carpentier classification type II is characterized by excessive motion of the mitral leaflet, accompanied by displacement of the free edge of one or both leaflets beyond the mitral annular plane (prolapse in myxomatous degeneration and tendinous chords or papillary muscle rupture).

Carpentier classification type IIIa implies restriction of movement of the leaflet during diastole and systole due to the shortening of the cords or the thickening of the leaflets, and the most frequent cause is rheumatic disease. This mechanism can also be objectified in the mitral annular calcification and drug-induced MR.

Carpentier classification type IIIb is characterized by restricted leaflet motion only in systole, secondary to leaflet tethering, displacement of papillary muscles, LV or LA dilatation, ventricular aneurism, or fibrosis. The paradigm of this group is MR related to ischemic cardiomyopathy.

It is remarkable that different etiological mechanisms can coexist in the same pathological valve. For instance, it is possible to find a valve with a posterior leaflet prolapse and, at the same time, with a systolic leaflet restriction. Hence, a coexistence of Carpentier type II and IIIb mechanisms would be described [7]. This combination of etiological mechanisms defines a mixed MR, which is more prevalent in elderly patients with fibrocalcific valvular changes and is gaining importance nowadays in Western countries. A cautious evaluation is required to elucidate the underlying mechanism. Frequently, there is a dominant alteration that will guide the treatment [13,14].

## 3. Severity Assessment in Practice

MR severity assessment is a challenging process in which a multimodality evaluation, integrating quantitative, semiquantitative and qualitative methods, as well as a detailed evaluation of the morphology and function of both LV and LA is the key [6]. There is a strong correlation between the hemodynamic status of the patient and the quantification of the MR. Quantification variability due to the regurgitation dependence on the cardiac afterload and preload makes it important to choose the right clinical time to perform a systematic and accurate echocardiographic assessment in a dynamic scenario [15,16]. In addition, this variability not only applies for the hemodynamic and volume status but also for the cardiac cycle. MR could be predominant in different stages of systole or even show a bimodal appearance subsidiary to the etiological mechanism, hence requiring a Doppler flow frame-to-frame integrative analysis [5]. Every parameter used to assess MR has its own limitations and pitfalls, making an integrative approach that analyzes all the available parameters overall essential to make a certain evaluation (Table 2).

Intervention over the mitral valve is only considered under the current practical guidelines if the regurgitation is considered severe, highlighting the accuracy of a proper evaluation [8].

### 3.1. Quantitative Parameters

MR should be evaluated by quantitative parameters along a continuous scale if possible [7]. Severity quantification derived from the Doppler color regurgitant flow visual assessment evaluating LA occupation and size is incorrect. The Doppler flow visual assessment should only be used to detect the MR; hence, the flow jet size and the MR are not only dependent on the MR severity but technical factors (gain, color flow scale settings) and the hemodynamic scenario. In this sense, for a similar MR severity, patients with high LA pressures, eccentric jets, or dilated LA will show a smaller flow area in front of those with normal LA size and pressures [17]. Notwithstanding, the detection of a great eccentric flow directed towards the posterior atrial wall are favorable for a severe MR. On the contrary, small and thin flows reaching right above the mitral valves are suggestive of mild MR [16].

**Figure 1:**
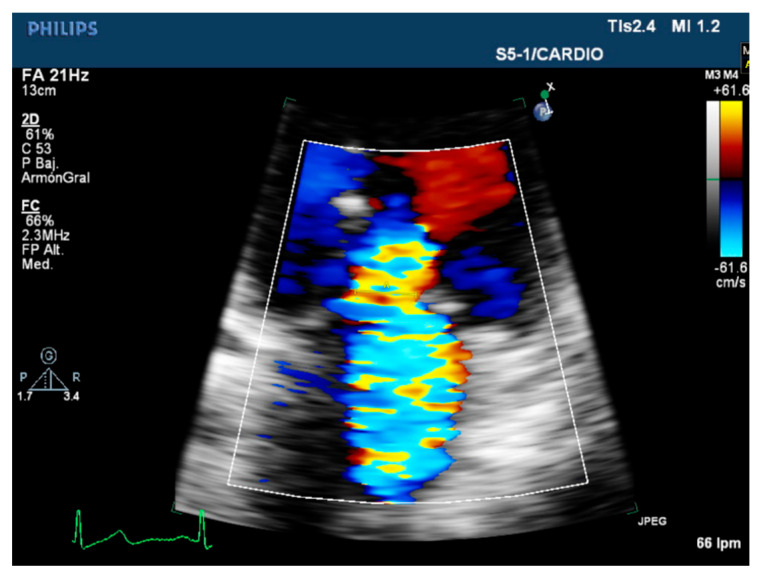
Transthoracic echocardiography. Vena contracta in severe mitral regurgitation.

A first approach to quantify the severity of the MR is the vena contracta (VC) measurement (Figure 1). The VC is the narrowest portion of the regurgitant jet at the level or just distal to the regurgitant orifice area (EROA) and reflects the basic diameter of the regurgitant office [8,18]. The measure of the VC should be performed perpendicular to the coaptation line, and it is necessary to appreciate the jet convergence surface, the VC, and the jet expansion to the LA in the same place. Attention should be paid to ensure a high frame rate and spatial resolution [3]. A VC value smaller than 3 mm suggests mild regurgitation and when the width exceeds 6 mm indicates severity. However, in secondary MR, a VC greater than 4 mm suggests severity. Intermediate values between 3 and 6 mm do not mean a moderate quantification but a need to perform other quantitative parameters [19]. When the MR is confirmed by several jets, the measurement of VC is not additive, and other methods should be used to assess severity in these cases [20]. Some limitations of this technique are the poor temporal resolution and the higher variability of the measures obtained from mild to moderate and a high inter-observer variability [18].

The VC parameter can be a 2D or 3D echocardiography-derived measurement. The 2D conventional color Doppler imaging does not provide an accurate orientation to obtain an appropriate cross-sectional view of the VC, as it could appear narrower in the four-chamber view and broader in the two-chamber view. The use of 3D echo formats, such as biplane imaging, to assess the VC in simultaneous orthogonal views increases the EROA evaluation accuracy, removing any geometrical assumption. This method is especially appropriate to reflect the effective EROA in elliptical orifices typically found in the secondary MR [21]. Nonetheless, the acquisition of a 3D data set is complex and time-consuming, and it is not available in all centers.

The color flow Doppler proximal isovelocity surface area (PISA) radius method is the most recommended quantitative approach when feasible to assess MR severity [17]. The PISA method is based in the principle and law of volume conservation. The regurgitant jet suffers a flow acceleration near the regurgitant orifice, generating series of isovelocity surfaces with hemispherical shape. Flow across any of this isovelocity hemispheric surfaces is equal to the flow through the regurgitant orifice, and for a given orifice, flow equals area of the orifice times velocity. Hence, flow that approaches the regurgitant orifice and passes through an isovelocity hemisphere is equal to flow that passes through the regurgitant orifice [22].

**Figure 2:**
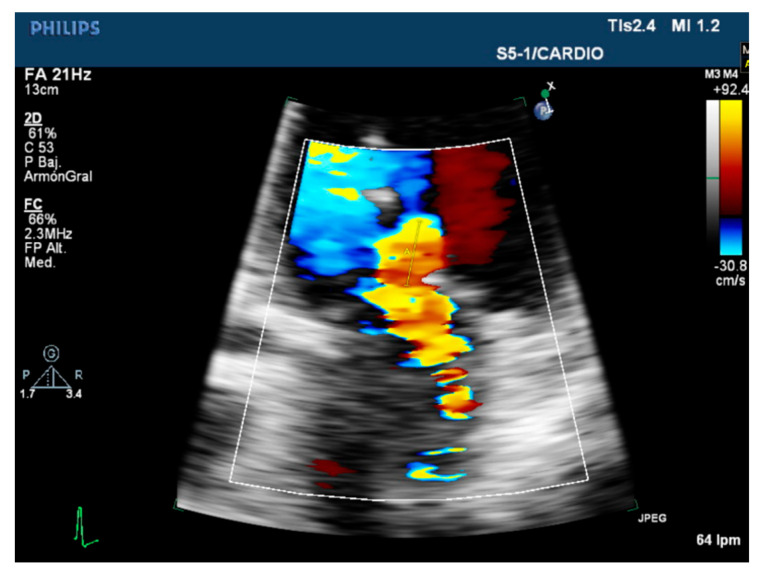
Transthoracic echocardiography. PISA measurement in severe mitral regurgitation.

For PISA measurement, the apical four-chamber plane is recommended; however, the parasternal long- or short-axis view is often useful for visualization of PISA in case of anterior valve prolapse [5]. The area of interest is optimized by lowering the imaging depth and reducing the Nyquist limit to approximately 15–40 cm/s. The radius of the PISA is measured at mid-systole using the first aliasing from a single-frame image. Even though this parameters provides a quantitative result, the presence of flow convergence at a higher Nyquist limit of 50–60 cm/s should draw attention to the existence of a severe MR (Figure 2) [16]. Optimization of the acquisition of this measure is essential, as any error of the radius will be squared when the EROA is obtained. Mistakes from 10% to 25% when measuring the radius have been seen among expert echocardiographists [23].

One of the notable limitations of this method is the time period in which this parameter is obtained given that it provides only an instantaneous regurgitant flow and area, as the integration of the radius measurement over the whole cycle is not technically possible nowadays. In this sense, the PISA measure could be variable thought the systole, decreasing, for instance, in mid-systole compared to early and late systole in secondary MR [24]. Compared to cardiac magnetic resonance quantification, PISA tends to overestimate the severity of the MR [25]. In addition, the PISA method assumes the hemispheric symmetry of the velocity distribution proximal to the circular regurgitant lesion, which may not be appropriate for eccentric, multiple jets, or elliptical regurgitant orifices. The geometry of PISA is dependent of the regurgitant orifice shape and surrounding MV leaflets morphology. For instance, in secondary MR, the PISA used to acquire an ellipsoidal shape and two or more different jets can be observed [16]. When the shape of the flow convergence zone is not hemisphere, this method can underestimate the MR severity, particularly if the ratio of long-axis length or short-axis length is superior to 1.5 [26]. On the other hand, the shape of the PISA in MR is rounder and could lead to underestimation of the EROA. These findings have derived an inferior threshold to define a severe secondary MR, which also needs to be assessed under optimal medical therapy [16].

Derived from the PISA measure, the EROA, regurgitant volume (RV), and regurgitant fraction (RF) calculation is available [5]. Their quantification is recommended, as they also show a prognostic value [27,28]. The regurgitant volume is the volume of blood that passes to the atrium through the regurgitant orifice in each cycle. It is directly proportional to the regurgitant orifice and is dependent on the pressures to both LA and LV. These determinations are derived from the Doppler color flow jet size determined by the blood density, orifice area, and velocity squared:

EROA = (2xπ × PISA^2^ × Aliasing Velocity) ÷ Peak Velocity of MR

MR is considered severe if EROA is superior to 40 mm^2^, and/or RV is equal or superior to 60 mL. Parallel to PISA severity threshold, the cut-off values of this parameter in secondary MR are significantly lower, being associated to poor prognosis if EROA is higher than 30 mm^2^, and RV superior to 45 mL [29].

This 2D PISA-derived measures loses accuracy when the PISA and the regurgitant orifice are not hemispherical, as frequently seen in the secondary MR, being more appropriate, in these cases, to consider the Doppler volumetric method to estimate the RV and RF [5]. Knowing the cross-sectional area of the mitral valve and the time velocity integral (VTI) of the flow that passes through the mitral valve in diastole and the cross-sectional area of the LV outflow tract and its VTI transmitral and transaortic stroke volume can be calculated. Applying the continuity equation, the difference between them is the RV [18]. This method is time-consuming, implies several measurements that can lead to miscalculation, and uses different cardiac cycles. In addition, it is not accurate if mild aortic regurgitation or intracardiac shunts are present. It should not be a first-line quantification method [17].

Inasmuch as the MR jet velocity increases, for instance, in aortic stenosis or systemic arterial hypertension, the EROA could overestimate the severity of the regurgitation with scarce velocity increments. Consequently, the EROA is also dependent on left-ventricular end diastolic pressure (LVEDP), gaining importance in the evaluation of secondary MR.

**Figure 3:**
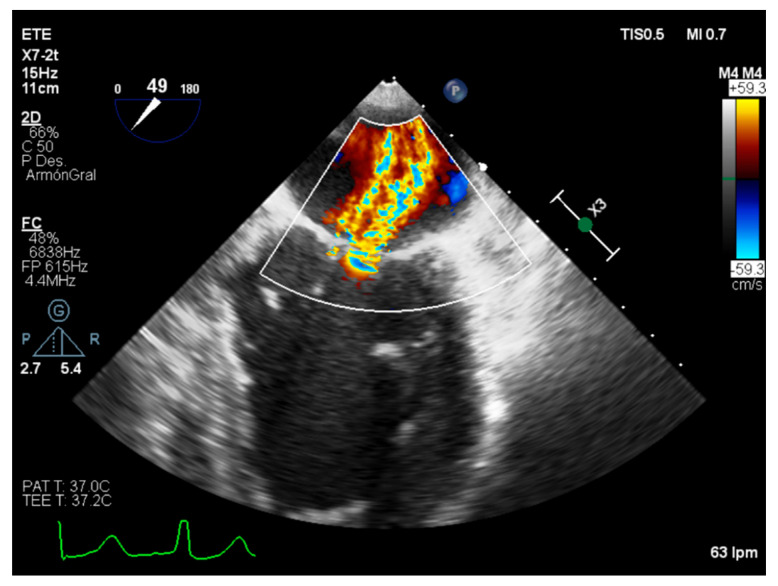
Transesophageal echocardiography. Mitral valve from Figure 4 with mild mitral regurgitation after transcatheter edge-to-edge repair procedure.

**Figure 4:**
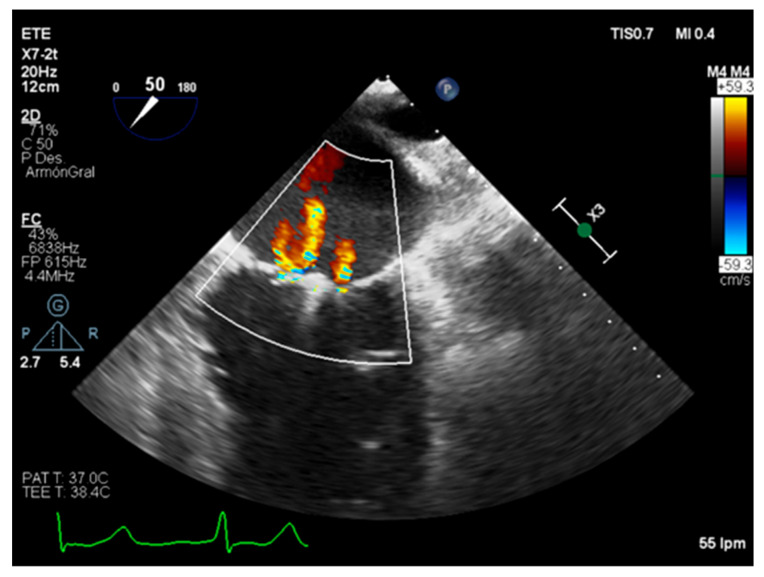
Transesophageal echocardiography. Disproportional mitral regurgitation in a non-ischemic dilated cardiomyopathy.

Recently, under the light of the COAPT and MITRA-FR trials, the characterization of secondary MR as proportional—patients with a MR degree proportional to its LVEDP and LV volume who are expected to respond to medical and resynchronization therapy—and disproportional—those whose MR is higher than the expected and can benefit from edge-to-edge mitral valve repair—has been proposed (Figure 3 and Figure 4) [30,31,32]. Even though the EROA, RV, and RF are strongly recommended as a first severity assessment approach, it is necessary to recognize its technical limitation and imprecision, corroborating its values with other findings [5].

### 3.2. Semiquantitative and Qualitative Parameters

Given the limitation and pitfalls of the quantitative parameters, it is recommended to analyze them together with qualitative observations that can reflect the hemodynamic consequences of the MR in the LV, LA, and the pulmonary circulation [6]. The mitral valve morphology, beyond being suggestive of a possible etiology, can sustain the severity quantification of the regurgitation if a flail leaflet or a ruptured chordae is appreciated. The evolution of the color in the MR should be used at first to mainly detect the MR but not to quantify its severity.

The color flow jet density, duration and direction, as well as the number of regurgitant jets observed can be useful, bearing in mind its tendency to overestimate or underestimate depending on the driving pressures and jet eccentricity. This parameter reflects the high systolic pressure gradient between the LV and LA [5]. Its continuous Doppler wave morphology, from faint and parabolic in mild regurgitation to dense and triangular when severe, can be useful as well [16]. Curiously, the velocity of the jet will not indicate the severity of the MR, but the intensity of the signal (a dense flow jet with a full envelope), which is considered a qualitative parameter of the MR, suggests severity [17]. Moreover, the color flow jet may show a notch, triangular morphology, and early peak velocity indicating elevated LA pressures. In eccentric MR, it may be difficult to record the whole wave [22].

The evaluation of the mitral inflow can rule out a severe regurgitation if there is an “A” wave dominance. On the contrary, in the absence of mitral stenosis, a restrictive pattern with high “E” waves greater than 1.2 m/s suggests severity. Conversely, a dominant A wave (reflecting atrial contraction) basically excludes severe MR [16]. Another simple method to assess the severity of MR is the mitral pulsed Doppler to aortic VTI ratio; the mitral inflow Doppler is obtained at the mitral leaflet tips and aortic flow at the annulus level in the apical four-chamber view. A VTI ratio higher than 1.4 strongly suggests severe MR, whereas a VTI ratio less than 1 is in favor of mild MR [33].

Additionally, the pulmonary vein flow pattern can complement the severity assessment. Normal pulmonary venous inflow shows forward inflow during both systole and diastole, with a brief flow reversal during atrial contraction. Pulmonary veins can be sampled in an apical four-chamber view [8]. The detection of systolic flow reversal is confirmatory of severe MR with a specificity of 92% [34]. Systolic blunting of pulmonary vein flow without systolic reversal is suggestive of significant MR; however, atrial fibrillation and elevated LA pressure from any cause can blunt forward systolic pulmonary vein flow.

Beyond the quantification of the regurgitant jet, it is essential to assess the LV and LA hemodynamic response to MR and record their evolutive measures and function [7]. MR imposes a volume overload in both LV and LA, leading to both chambers’ dilatation and an increase in the LA and pulmonary pressures. If this volume overload is acute, LV and LA might not be dilated yet, but they can show elevated pressures [14]. In chronic MR, the continuous volume overload can result in a progressive deterioration of ventricular function besides the chamber dilatation, as this is a determinant to consider intervention over the mitral valve [9]. In addition, the interaction between both chambers’ hemodynamics is complex and has a strong influence on the final regurgitation quantification. For instance, a stiff and dilated LA with elevated basal pressures can lead to underestimation if all data available from LA and LV geometrics and function are not accurately evaluated.

### 3.3. Role of Stress Echocardiography

Exercise intolerance and appearance of the symptoms, essentially dyspnea, with physical efforts constitute a Class I indication for intervention in patients with primary severe MR [35]. Stress echocardiography, which can be performed with exercise testing or dobutamine infusion, allows unmasking severe regurgitation as well as assessing LV and right ventricle performance and pulmonary pressure under stress conditions [36]. The appreciation of an MR severity increment, a lack of ventricular contractile reserve, and a systolic pulmonary pressure rise over 60 mmHg are predictive of worse prognosis, symptoms progression, and need for an intervention [37]. Particularly in the ischemic population, characterized by eccentric and variable regurgitant flows, stress echocardiography has a especial interest in patients with dyspnea out of proportion with the severity of their resting LV performance and MR severity, those whom have suffered acute pulmonary edema without a clear pathophysiological mechanism, and in the peri-surgical time, before assessing a need to mitral intervention when revascularization is planned and following surgery to identify persistence of pulmonary arterial hypertension [38]. On the other hand, secondary MR has also demonstrated a dynamic behavior during exercise and stress echocardiography, providing useful information to predict outcomes and plan further therapeutical options in these patients, such as cardiac resynchronization or transcatheter edge-to-edge repair [39].

## 4. Assessment of Suitability of Valvular Repair

If there is an indication for surgery, mitral valve repair should be considered as a first choice if feasible since it has shown better survival rates [7,17]. A transesophageal echocardiography evaluation on morphology and etiology is necessary prior to every technique consideration. The paradigm of valve abnormality leading to a successful repair is the Carpentier type II, especially when involving one scallop of the posterior leaflet [7]. In primary MR, there are few echocardiographic findings that suggest a high probability of significant MR after a valve repair: the presence of a large central jet, annular diameter higher than 50 mm, involvement of three or more scallops, substantial valve and ring calcification, and lack of valve tissue, for instance, after suffering and infectious endocarditis [10].

Transcatheter edge-to-edge repair may be considered in those symptomatic patients with high surgical risk [39]. Findings of a perforated leaflet, lack of primary and secondary chordal support, severe calcification of the grasping area, significant mitral stenosis, short posterior leaflet (shorter than 7 mm), systolic and diastolic restriction typical of rheumatic valve disease, and the presence of a gap superior to 2 mm between leaflets makes the mitral valve unsuitable for edge-to-edge repair [40].

## 5. Role of Intraoperative Echocardiography

**Figure 5:**
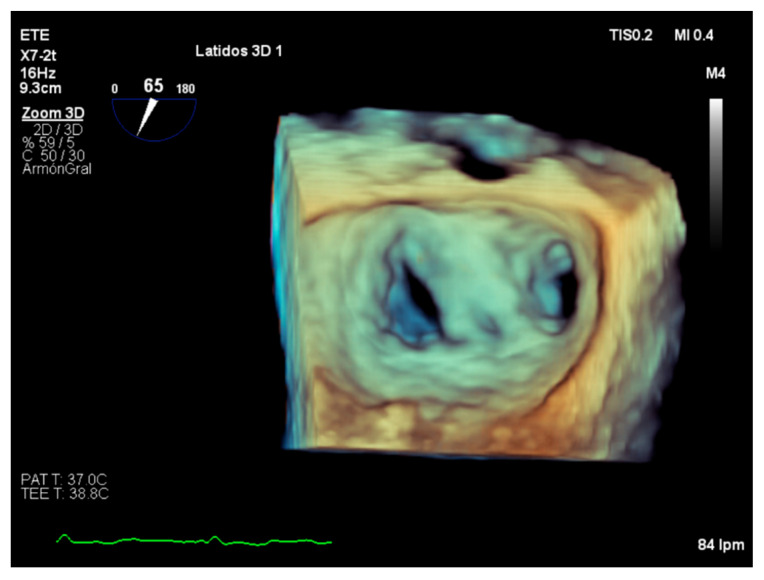
3D transesophageal echocardiography. Clips placed on the mitral valve after transcatheter edge-to-edge repair procedure.

Intraoperative transesophageal echocardiography is a basic component of contemporary cardiac surgery planning and results evaluation, allowing the detection of suboptimal results inside the operating room [41]. In addition, assessment of the results and follow-up evaluation is essential in the transcatheter edge-to-edge guiding procedure (Figure 5) [42].

## 6. Future Research Directions

MR assessment is a growing clinical challenge in which pathophysiology, clinical presentation, imaging assessment, and therapeutical options differ as to whether their etiology is primary or secondary and, finally, if their origin is ischemic or non-ischemic. Echocardiography allows a differential diagnosis between these entities and allows timing and guidance for therapeutical procedures. Nonetheless, there is a wide range of limitations on the echocardiographic parameters based on pathophysiological principles, which forces us to make an integration of all the clinical and multimodality imaging data to make clinical decisions every day. The results of the actual and future randomized clinical trials in the field of percutaneous interventionism should be carefully evaluated, as they could guide future severity thresholds and poor prognosis markers. The innovation in other imaging techniques, such as cardiac magnetic resonance and cardiac tomography, will lead to a day-to day integration of different imaging studies to make an accurate evaluation.

## 7. Conclusions

The. mitral valve is a complex anatomical structure whose function depends on the integrity of its structural components as well as the LA and LV geometry and function. The assessment of the regurgitation mechanism and its severity has strong implications on therapeutic management and prognosis. Although the quantitative parameters should be the first-line approach, it is remarkable that, due to its limitations and pitfalls, other qualitative and semiquantitative methods should complement the evaluation. The combination of transthoracic and transesophageal echocardiography allows a more precise and detailed evaluation. Additionally, if there is a dissociation between the clinics and the echocardiographic findings, a stress echocardiography to unmask a potential severe regurgitation should be considered. Moreover, the suitability of repair should always be considered.

## Figures and Tables

**Table 1:** Carpentier’s classification.

Carpentier’s Classification	Leaflets Motion	Anatomical Session	Etiologies
Type I	Normal.	Leaflet perforation. Annular dilatation.	Degenerative (annular calcification), infectious endocarditis, inflammatory, congenital cleft defect.
Type II	Excesive.	Chordal rupture. Chordal elongation. Papillary muscle rupture.	Degenerative (Barlow’s disease), congenital, infectious, ischemic.
Type IIIa	Restricted in both systole and diastole.	Commisural or chordal fussion. Leaflet thickening. Leaflet calcification.	Rheumatic, inflammatory, radiation, drugs.
Type IIIb	Restricted in systole.	Ventricular dilatation. Chordal thickening or shortening.	Ischemic and non-ischemic.

**Table 2:** Integration of different severity assessment indexes. EROA (regurgitant orifice area), RV (regurgitant volume), TVI (time velocity integral), MV (mitral valve).

Parameters	Type	Indicatives of Mild MR	Indicatives of Severe MR
**EROA (mm^2^)**	Quantitative.	<20	≥40
**RV**	Quantitative.	<30	≥60
**Vena contracta width (mm)**	Semi-quantitative. Quantitative in 3D performance.	<3	≥7 (≥8 for biplane)
**Mitral inflow**	Semi-quantitative.	A wave dominance.	E wave dominance.
**TVI mitral/TVI aortic**	Semi-quantitative.	<1	<1.4
**Pulmonary vein inflow**	Semi-quantitative.	Systolic dominance.	Systolic flow reversal.
**MV morphology**	Qualitative.	Normal/abnormal.	Flail leaflet or ruptured papillary muscle.
**Color flow jet**	Qualitative.	Small, central.	Large central jet or eccentric reaching the posterior LA wall.
**Continuous doppler wave morphology.**	Qualitative.	Faint/parabolic.	Dense/triangular.

## Data Availability

Not applicable.
